# Hyodeoxycholic acid modulates gut microbiota and bile acid metabolism to enhance intestinal barrier function in piglets

**DOI:** 10.3389/fvets.2025.1610956

**Published:** 2025-06-20

**Authors:** Jie Chong, Yongming Zhou, Zhi Li, Xiaokai Li, Jinwei Zhang, Haoran Cao, Jideng Ma, Liangpeng Ge, Hang Zhong, Jing Sun

**Affiliations:** ^1^Chongqing Academy of Animal Sciences, Chongqing, China; ^2^Shenzhen Academy of Metrology & Quality Inspection, Shenzhen, China; ^3^National Center of Technology Innovation for Pigs, Chongqing, China; ^4^Farm Animal Genetic Resources Exploration and Innovation Key Laboratory of Sichuan Province, Sichuan Agricultural University, Chengdu, China

**Keywords:** hyodeoxycholic acid, gut microbiota, intestinal barrier function, bile acid metabolism, TGR5 signal pathway, piglets

## Abstract

Oral bile acids, particularly hyodeoxycholic acid (HDCA), serve as critical drivers for gut microbial community maturation in mice. In the first study, Cy5-labeled HDCA combined with fluorescence imaging revealed rapid gastrointestinal transit of HDCA in piglets, contrasting with its delayed absorption observed in mice. In the second study, the effects of the oral HDCA supplementation on microbiota-host metabolic interactions were investigated using four piglet model groups: OPM-HDCA (naturally born, raised germ-free (GF), and orally administered HDCA), OPM-CON (naturally born, raised GF, and orally administered PBS), SPF-HDCA (naturally born, raised GF, and received fecal microbiota transplantation (FMT) and HDCA), and SPF-CON (naturally born, raised GF with FMT but no HDCA). The results demonstrated that HDCA administration at 0.2 mg/mL suppressed body weight gain in piglets, which was alleviated by FMT. HDCA significantly altered gut microbiota composition in SPF piglets, markedly increasing the *Lactobacillus* abundance (37.97% vs. 5.28% in SPF-CON) while decreasing the proportion of *Streptococcus* (28.34% vs. 38.65%) and pathogenic family *Erysipelotrichaceae* (0.35% vs. 17.15%). Concurrently, HDCA enhanced intestinal barrier integrity by upregulating tight junction proteins (ZO-1, Claudin, Occludin) and suppressing pro-inflammatory cytokines (TNF-*α*, IL-1β). Additionally, HDCA significantly upregulated ileal gene expression of *CYP7A1* (cytochrome P450 family 7 subfamily A member 1) and *TGR5* (G protein-coupled bile acid receptor 1) in both SPF-HDCA and OPM-HDCA groups compared to their respective controls (*p* < 0.05). These findings demonstrate that HDCA exerts microbiota-dependent effects on growth performance, intestinal barrier function, and bile acid metabolism in piglets. Although 0.2 mg/mL HDCA treatment suppressed body weight gain, it potentially enhanced intestinal barrier integrity by activating the *TGR5* signaling pathway and increasing the abundance of beneficial bacteria such as Lactobacillus. These results also highlight the critical role of early-life gut microbiota in nutritional interventions, providing a basis for developing precision nutritional strategies targeting intestinal microbial ecology in piglets.

## 1 Introduction

Bile acids (BAs), classically known for their role in lipid digestion and cholesterol homeostasis, have emerged as critical signaling molecules regulating metabolic and immune functions in animals (1). Recent studies highlight hyocholic acid and its derivatives (HCAs) as promising biomarkers and therapeutic agents for treating non-alcoholic fatty liver disease (NAFLD) (2), regulating blood glucose (3), metabolic disorders (4), and alleviating colitis (5), with its efficacy demonstrated in mouse studies. HCAs are found in trace amounts in human blood but account for over 70% of total BA pool in pigs (6), exhibiting unique species-specific metabolic profiles compared to other mammals and poultry (7). One of the important microbial-derived secondary bile acids in HCAs, hyodeoxycholic acid (HDCA), can indirectly affect the physiological functions of the host by regulating the structure of the intestinal microbial community and its metabolites. Studies have shown that HDCA can enhance the intestinal epithelial integrity and immune response ability in weaned piglets (8), effectively alleviating lipopolysaccharide-induced intestinal inflammation in piglets (9). In addition, HDCA also potentially improves the feed efficiency and of growing-finishing pigs (10), reduce the backfat thickness (11), and thereby improve the carcass quality of the finishing pigs. However, the absorption kinetics, microbiota-dependent metabolic effects, and physiological impacts of exogenous HDCA in newborn piglets remain poorly understood, limiting its application in precision swine nutrition.

The gut microbial community greatly changes in the early life, which play a critical role in shaping infant health and the subsequent physiological functions of the host. The interplay between BAs and gut microbiota is pivotal for host health. Gut microbes extensively modify BAs through deconjugation, dehydrogenation, and 7α-dehydroxylation, generating secondary BAs that reciprocally shape microbial composition (12, 13). For instance, cholic acid supplementation enriches *Clostridium* and *Erysipelotrichaceae* in rodents (14), while dysbiosis in irritable bowel syndrome patients correlates with elevated primary BAs (15, 16). Research has demonstrated that oral bile acids serve as a key driving factor for the maturation of the intestinal microbial community during the neonatal period. Furthermore, the early metabolic profile of the intestine is dynamically modulated during the colonization process of the intestinal microbiota, with this modulation being influenced by nutritional factors (e.g., breast milk or formula milk) (17) and birth modes (e.g., cesarean delivery or vaginal delivery) (18). However, limited information is available regarding how the colonization of early microbiota and bile acid (such as HDCA) jointly influence postnatal growth of piglets, intestinal health, and the maturation of bile acid metabolism. This study aimed to elucidate the effects and underlying mechanisms of the combined action between gut microbiota and HDCA on growth performance and intestinal health in suckling piglets.

## 2 Materials and methods

### 2.1 Ethics approval

All procedures adhered to ARRIVE guidelines and were approved by the Chongqing Academy of Animal Sciences (mouse experiment approval number: Cqaa-2023007; pig experiment approval number: Cqaa-2023006). Female germ-free C57BL/6 J mice used in this study were supplied by GemPharmatech Co., Ltd. (Jiangsu, Chian), while the experimental pigs were provided by the Experimental Pig Engineering Center of the Chongqing Academy of Animal Science (Chongqing, China).

### 2.2 Animals

To visually compare the impact of host species differences on HDCA absorption kinetics, eight newborn piglets (littermates with minimal microbial colonization at birth, approximating germ-free conditions, birth weight: 904 ± 161 g) and eight 6- to 8-week-old C57BL/6 J germ-free mice (body weight: 21.6 ± 1.2 g) were used for cross-species HDCA tracking.

To investigate the combined effects of early intestinal microbiota colonization and HDCA on the postnatal growth, intestinal health, and bile acid metabolic profile of piglets, we developed the following animal models for this study. In detail, 20 newborn Rongchang piglets were born through natural delivery, they were immediately transferred to Class III isolators (CBC Inc., USA). All piglets were fed *γ*-irradiated milk replacer (25 kGy; milk powder: sterile water = 1:4) five times daily in positive-pressure isolators and were reared under identical feeding management conditions (28 ± 1°C, 40–70% humidity) until sampling for slaughter. Ten newborn piglets were randomly assigned to two groups: the SPF-HDCA group (*n* = 5, birth weight: 1211 ± 235 g) and the SPF-CON group (*n* = 5, birth weight: 1375 ± 485 g). Each piglet in the SPF-HDCA group received an oral HDCA solution (0.2 mg/mL in PBS, 0.5 mL/day; 0.22 μm filtered) daily and received fecal microbiota transplantation (FMT) with fecal suspension from a healthy SPF-grade 1-month-old piglet (concentration: 1 × 10^8^ CFU/mL, 0.5 mL/piglet per day) for three consecutive days. The SPF-CON group underwent the same FMT protocol but received an equivalent volume of sterilized PBS solution orally as a control. Another 10 newborn piglets were randomly assigned to the OPM-HDCA (n = 5, birth weight: 616 ± 172 g) and OPM-CON group (*n* = 5, 742 ± 308 g). Each piglet in the OPM-HDCA group received the oral HDCA solution (0.2 mg/mL in PBS, 0.5 mL/day; 0.22 μm filtered) daily but did not undergo FMT, maintaining an “oligo-microbiota state.” The OPM-CON group received only the equivalent volume of sterilized PBS solution orally as a control and did not undergo FMT.

### 2.3 Cross-species HDCA tracking in pig and mouse models

To characterize HDCA absorption dynamics, 8 SPF newborn piglets received oral gavage of Cy5-labeled HDCA (0.2 mg/mL, 1 mL per piglet). Fluorescence imaging (PerkinElmer IVIS Lumina III, USA) was performed at 1, 2, 2.5, 3, 3.5, 5, 5.5, and 6 h post-administration (*n* = 1 piglet per time point). Following anesthesia with isoflurane and humane euthanasia via intravenous sodium pentobarbital, intact gastrointestinal tracts were excised and imaged within the 640–710 nm emission range. Regional fluorescence intensity (photons/s/cm^2^/sr normalized to μW/cm^2^) was quantified using Living Image® software (PerkinElmer Inc., USA). Additionally, eight 6- to 8-week-old C57BL/6 J germ-free mice received Cy5-HDCA (0.2 mg/mL, 1 mL per mouse) via oral gavage. *In vivo* and ex vivo imaging was conducted at 5 min, 1, 6, 18, 24, 48, 56, and 72 h post-administration (*n* = 1 per time point). Mice were anesthetized with CO_2_, euthanized by cervical dislocation, and their gastrointestinal tracts were imaged as described above.

### 2.4 Sample collection

In the second experiment, we explored the combined effects of oral HDCA supplementation and FMT on microbiota-host metabolic interactions in piglets. The piglets were reared until they reached 21 days of age. Following weighing, blood samples were collected under isoflurane anesthesia from the anterior vena cava. The blood samples were divided into three portions: one tube was placed in a heparin anticoagulant tube for blood routine analysis, another tube was placed in an EDTA anticoagulant tube for biochemical index measurements, and the remaining blood was used for separating the serum and was stored at −80°C. The piglets were euthanized by exsanguination under anesthesia. The abdominal cavity was opened on the sampling platform, and bile was collected. The heart, liver, spleen, lung, and kidney tissues were excised and weighed. Liver tissue was snap-frozen in liquid nitrogen and stored at −80°C. Small intestine tissues (ileum and jejunum) were isolated; a 1-cm segment was fixed in 4% paraformaldehyde for histomorphological analysis, while the remaining small intestine and colon tissues were rinsed with PBS, rapidly frozen in liquid nitrogen, and stored at −80°C.

### 2.5 Blood parameters analysis

Twenty-three hematological and 24 biochemical parameters were analyzed (Supplementary Tables S3, S4). The levels of inflammatory markers, including tumor necrosis factor-alpha (TNF-*α*), interleukin-1 (IL-1β), and interleukin-6 (IL-6), as well as metabolic indicators, such as alanine aminotransferase (ALT), aspartate aminotransferase (AST), were quantified using the ELISA method (Shanghai Yuanxin Biotechnology Co. Ltd., China).

### 2.6 16S rRNA amplicon sequencing and data analysis

DNA extraction, PCR amplification, and DNA quantification by the standardized protocol of Shanghai Majorbio Bio-pharm Technology (Shanghai, China). Briefly, DNA was extracted from the ileum contents using the Qiagen DNA isolation kit (Qiagen, Hilden, Germany) and followed by the manufacturer’s instructions. The V3-V4 hypervariable region of the 16S bacterial rRNA was amplified using primers 338F (5’-ACTCCTACGGGAGGCAGCAG-3′) and 806R (5’-GGACTACHVGGGTWTCTAAT-3′). The PCR products were extracted from 2% agarose gel and purified using the AxyPre DNA Gel Extraction Kit (Axygen, USA) according to the manufacturer’s instructions and quantified using the QuantiFlour™-ST Blue Fluorescence Quantitation System (Promega, USA).

Purified amplicons were pooled in equimolar amounts and paired-end sequenced on an Illumina Nextseq2000 platform (Illumina, San Diego, USA) according to the standard protocols by Majorbio Bio-Pharm Technology Co. Ltd. (Shanghai, China). Raw illumina fastq files were demultiplexed, quality filtered with fastp (v0.19.6) and merged with FLASH (v1.2.11). Then the high-quality sequences were de-noised using DADA2 plugin in the Qiime2^1^ pipeline with recommended parameters, which obtains single nucleotide resolution based on error profiles within samples. Taxonomic assignment of amplicon sequence variants (ASVs) was performed using the Naïve Bayes consensus taxonomy classifier implemented in Qiime2 and SILVA 16S rRNA database (v138). The metagenomic function was predicted by PICRUSt2 (Phylogenetic Investigation of Communities by Reconstruction of Unobserved States, v2.2.0) based on ASV representative sequences.

### 2.7 Hematoxylin–eosin (H&E) staining

Jejunal and ileal specimens were fixed with 4% paraformaldehyde (PFA). PFA-fixed specimens were embedded in paraffin, sectioned (4 mm), and routinely stained with hematoxylin–eosin (H&E, Beyotime, China). The stained sections were scanned using a Pannoramic 250 scanner (3DHISTECH, Hungary), and the height of villi and the depth of crypts were measured on the tissue sections using the ImageJ software (National Institutes of Health, USA).

### 2.8 Analysis of intestinal permeability indicators and bile acid profiles

Serum levels of endotoxin (LPS), diamine oxidase (DAO), immunoglobulin G (IgG), and ileal tight junction proteins (Occludin, Claudin, tight junction protein 1 (ZO-1)) were quantified using ELISA kits (Beyotime Biotechnology Co. Ltd., Shanghai, China). Targeted metabolomics analysis was conducted on liver, bile, and intestinal bile acid (BA) profiles, measuring 47 analytes including cholic acid (CA), HDCA, chenodeoxycholic acid (CDCA) by liquid chromatography-mass spectrometry (LC–MS) (specific measurement indicators are detailed in Supplementary Table S2), as previously described by Krautbauer and Liebisch (19).

### 2.9 Total RNA extraction and RT-qPCR fluorescence quantitative analysis

Total RNA was extracted from the liver, ileum and colon tissues of piglets using the HiPure Universal RNA Mini Kit (MagGenome, China). The RNA quality of the extracted RNA was assessed using a NanoDrop 2000 spectrophotometer (Thermo Fisher Scientific Inc., USA). Qualified RNA samples were reverse transcribed into cDNA according to the instructions provided in the Promega GoScript Reverse Transcription System kit (Promega Corporation Inc., USA). Primer pairs were designed using Primer Premier 5.0 software (Premier Biosoft International Inc., USA) and synthesized by Suzhou Jinweizhi Biotechnoloy Co., Ltd. (details are presented in Supplementary Table S1), with *GAPDH* serving as the housekeeping gene. RT-qPCR analysis was conducted using the TB Green® Premix Ex Taq™ II kit (TaKaRa, Japan). The thermal cycling program consisted of an initial denaturation step at 95°C for 30 s (1 cycle), followed by 40 cycles of 95°C for 5 s and 60°C for 34 s, and a final dissociation curve analysis step involving 95°C for 15 s, 60°C for 1 min, 95°C for 15 s (1 cycle). The results were normalized to the CT value of *GAPDH*, and the relative expression levels of the genes was calculated using the 2^-ΔΔCT^ method.

### 2.10 Statistical analysis

The effects of HDCA treatment were analyzed separately within the SPF (SPF-HDCA vs. SPF-CON) and OPM (OPM-HDCA vs. OPM-CON) groups, without cross-comparison between the groups, to elucidate the specific impact of HDCA treatment under distinct gut microbiota backgrounds. Separate analyses were conducted to address the substantial differences in variance within the SPF and OPM groups and avoid potential statistical biases. Data on the ratio of villus height to crypt depth, ileal tight junction proteins, pro-inflammatory cytokines, serum LPS, DAO and IgG, concentration of bile acids, hematological parameters, and bacterial *α*-diversity indices (Shannon, and ACE), and gene expression were analyzed by the Kruskal-Wallis H test and the false discovery rate (FDR) multiple comparison method. The Gut Microbiota Health Index (GMHI), quantified using the Shannon diversity index, was analyzed using the Mann–Whitney U test with two-tailed testing. The Benjamini-Hochberg false discovery rate (FDR) correction was applied, with a corrected *p*-value < 0.05 considered statistically significant. Data visualization was conducted using GraphPad Prism 9.0 (GraphPad Software, San Diego, CA, USA). Results are presented as mean ± standard error of the mean (SEM). Statistical Significance: *p* < 0.05, *p < 0.01, **p < 0.001.

## 3 Results

### 3.1 Species-specific HDCA absorption dynamics

**Figure 1:**
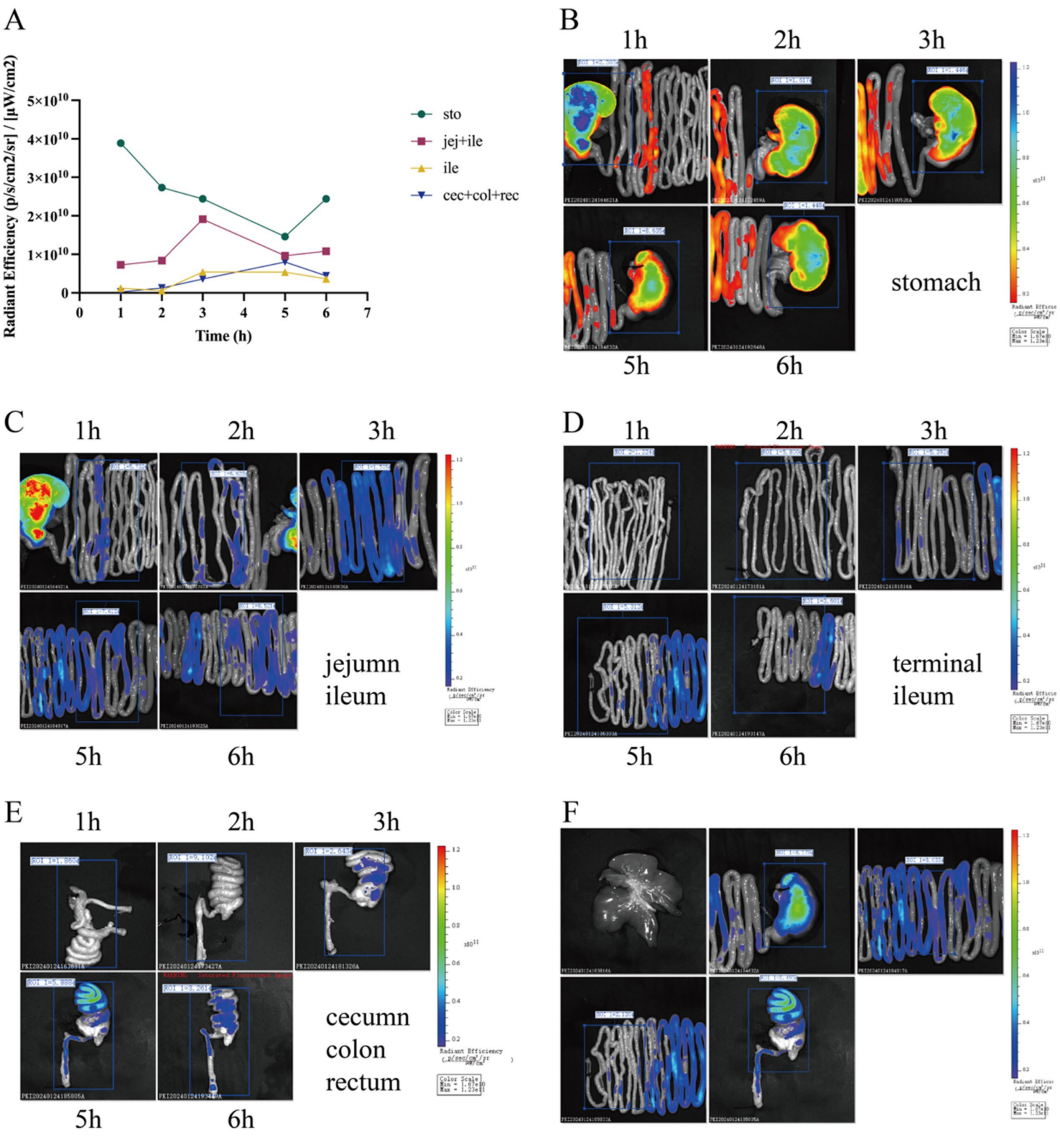
Tracking of HDCA absorption in piglets using Cy5 fluorescent dye. **(A)** Line graph depicting fluorescence intensity at various time points (1, 2, 2.5, 3, 3.5, 5, 5.5, and 6 h) across different regions of the gastrointestinal tract, including the stomach, jejunum, proximal ileum, distal ileum, cecum, colon, and rectum. **(B)** Fluorescence imaging of the stomach at different time intervals. **(C)** Fluorescence imaging of the jejunum and proximal ileum at specified time intervals. **(D)** Fluorescence imaging of the distal ileum at specified time intervals. **(E)** Fluorescence imaging of the colon at specific time intervals. **(F)** Fluorescence imaging of piglets at the 5-h time point.

**Figure 2:**
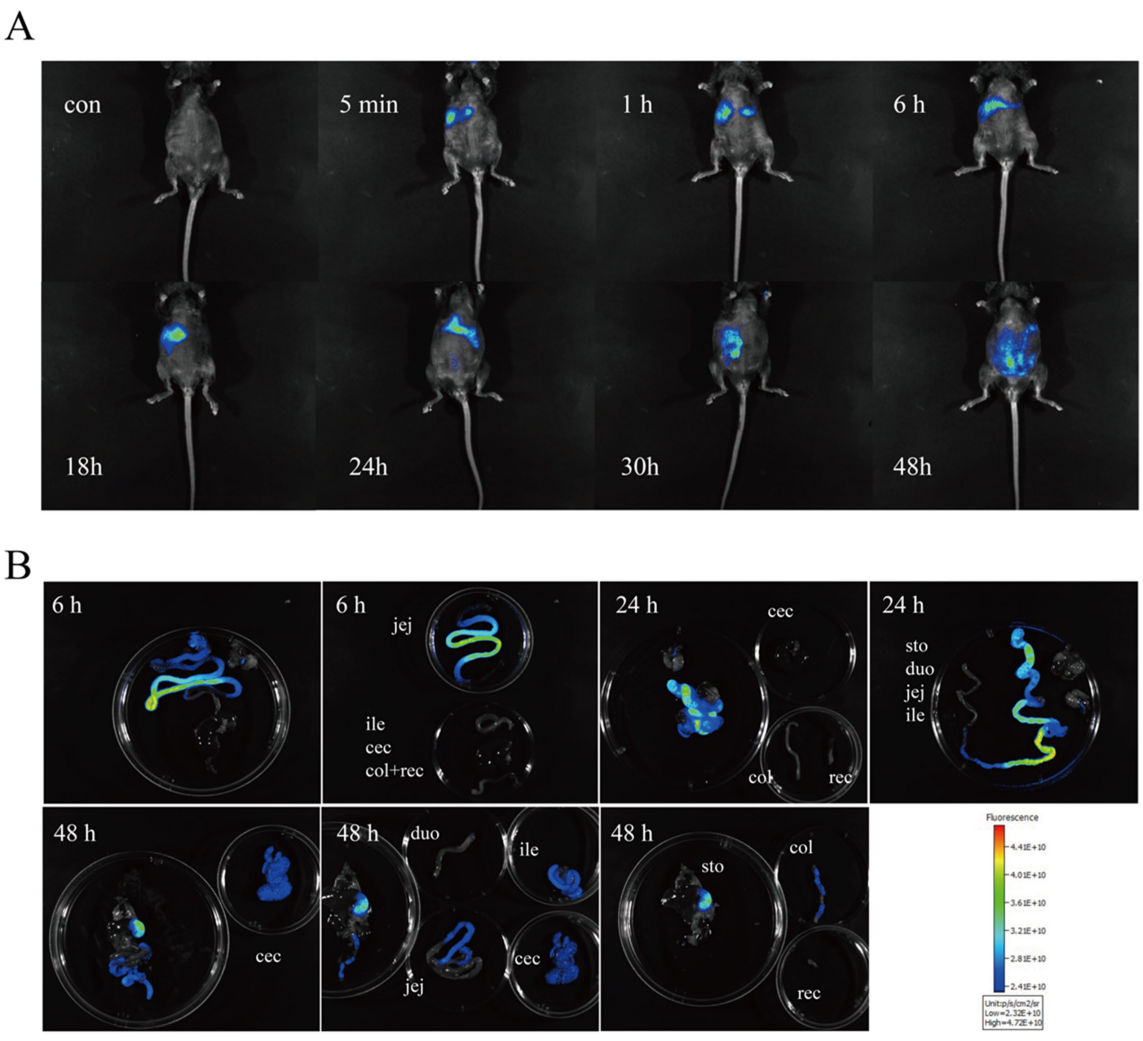
Monitoring the absorption of HDCA in germ-free mice using Cy5 fluorescent Dye. **(A)** *In vivo* imaging of GF mice at 0, 5 min, 1 h, 6 h, 18 h, 24 h, 30 h, and 48 h post-gavage with HDCA. **(B)** Ex vivo imaging of various intestinal segments at 6 h, 24 h, and 48 h post-gavage.

In the first experiment, fluorescence tracking revealed distinct HDCA transit patterns between newborn piglets and germ-free mice (Figures 1, 2). In piglets, Cy5-HDCA rapidly migrated from the stomach to the proximal jejunum within 1 h (fluorescence intensity: 0.73 × 10^10^ photons/s/cm^2^/sr per μW/cm^2^), peaking in the jejunum/ileum at 3 h (0.96 × 10^10^ photons/s/cm^2^/sr per μW/cm^2^) and completing excretion by 6 h. In contrast, germ-free mice retained HDCA in the stomach for > 1 h, with delayed transit to the ileum (24 h) and colon (48 h) (*p* < 0.001 vs. piglets).

### 3.2 Body weight gain and organosomatic indices

In the second experiment, continuous oral HDCA administration significantly reduced the mean body weight gain in OPM piglets (1.78 ± 0.24 kg in OPM-HDCA group vs. 2.17 ± 0.33 kg in OPM-CON group, *p* < 0.05) and also decreased the mean body weight gain in SPF piglets (2.82 ± 0.29 kg in SPF-HDCA group vs. 3.13 ± 0.30 kg in SPF-CON group, *p* > 0.05).

Compared to OPM-CON group, the OPM-HDCA group showed slightly lower pulmonary index (lung-to-body weight ratio) and renal index (kidney-to-body weight ratio) (*p* > 0.05, Table 1) but slightly higher cardiac index (heart-to-body weight ratio), hepatic index (liver-to-body weight ratio), and splenic index (spleen-to-body weight ratio) (*p* > 0.05). Similarly, oral HDCA treatment slightly reduced the renal index (*p* > 0.05) but increased cardiac index, hepatic index, splenic index, and pulmonary index (*p* > 0.05), indicating no HDCA-induced developmental toxicity in this study.

**Table 1:** The impact of HDCA treatment on organ development in piglets^1^^,^^2^.

Organ index (%)	OPM-CON group	OPM-HDCA group	*p*-value (OPM-CON vs. OPM-HDCA)	SPF-CON group	SPF-HDCA group	*p*-value (SPF-CON vs. SPF-HDCA)
Cardiac Index	0.63 ± 0.03	0.65 ± 0.01	0.48	0.57 ± 0.02	0.63 ± 0.02	0.03
Hepatic Index	2.96 ± 0.19	3.15 ± 0.08	0.39	4.42 ± 0.23	4.56 ± 0.26	0.68
Splenic Index	0.24 ± 0.01	0.27 ± 0.01	0.15	0.24 ± 0.02	0.25 ± 0.02	0.79
Pulmonary Index	1.35 ± 0.09	1.21 ± 0.06	0.22	1.31 ± 0.06	1.37 ± 0.06	0.50
Renal Index	0.78 ± 0.04	0.76 ± 0.02	0.78	0.79 ± 0.02	0.75 ± 0.03	0.17

1 Cardiac index: calculated as heart-to-body weight ratio; Hepatic index: calculated as liver-to-body weight ratio; Splenic index: calculated as spleen-to-body weight ratio; Pulmonary index: calculated as lung-to-body weight ratio; Renal index: calculated as kidney-to-body weight ratio. Data is represented by Mean ± SEM, *n* = 5/group.

2 SPF-HDCA group: piglets born naturally, raised in a germ-free environment, subsequently received fecal microbiota transplantation (FMT), and received 0.2 mg/mL HDCA orally; SPF-CON group: group: piglets born naturally, raised in a germ-free environment, subsequently received fecal microbiota transplantation (FMT), and received the equivalent volume of sterilized PBS solution orally as a control; OPM-HDCA group: piglets born naturally, raised in a germ-free environment without undergoing FMT, received 0.2 mg/mL HDCA orally; OPM-CON group: piglets born naturally, raised in a germ-free environment without undergoing FMT, received the equivalent volume of sterilized PBS solution orally as a control.

### 3.3 Hematological and biochemical profiling

In the second experiment, continuous oral administration of HDCA significantly altered hematological and serum biochemical parameters. In the OPM-HDCA group, red blood cell count (RBC) increased from 4.92 × 10^9^/L to 5.73 × 10^9^/L, and hematocrit (HCT) rose from 26.48 to 31.37% compared to the OPM-CON group (*p* < 0.05). These changes were more pronounced in the SPF-HDCA group that received FMT treatment. Compared to the SPF-CON group, neutrophil count and percentage decreased, while lymphocyte percentage and corpuscular hemoglobin concentration increased significantly (*p* < 0.05, Supplementary Table S3). Additionally, compared to the SPF-CON group, the SPF-HDCA group showed a significant increase in serum levels of 15 biochemical indicators, including albumin, total protein, globulin, total bilirubin, gamma-glutamyl transferase, aspartate aminotransferase, alanine aminotransferase, lipase, lactate dehydrogenase, creatine kinase, creatinine, urea, urine protein-to-creatinine ratio, calcium, and inorganic phosphorus (*p* < 0.05, Supplementary Table S4).

### 3.4 Microbial community analysis

**Figure 3:**
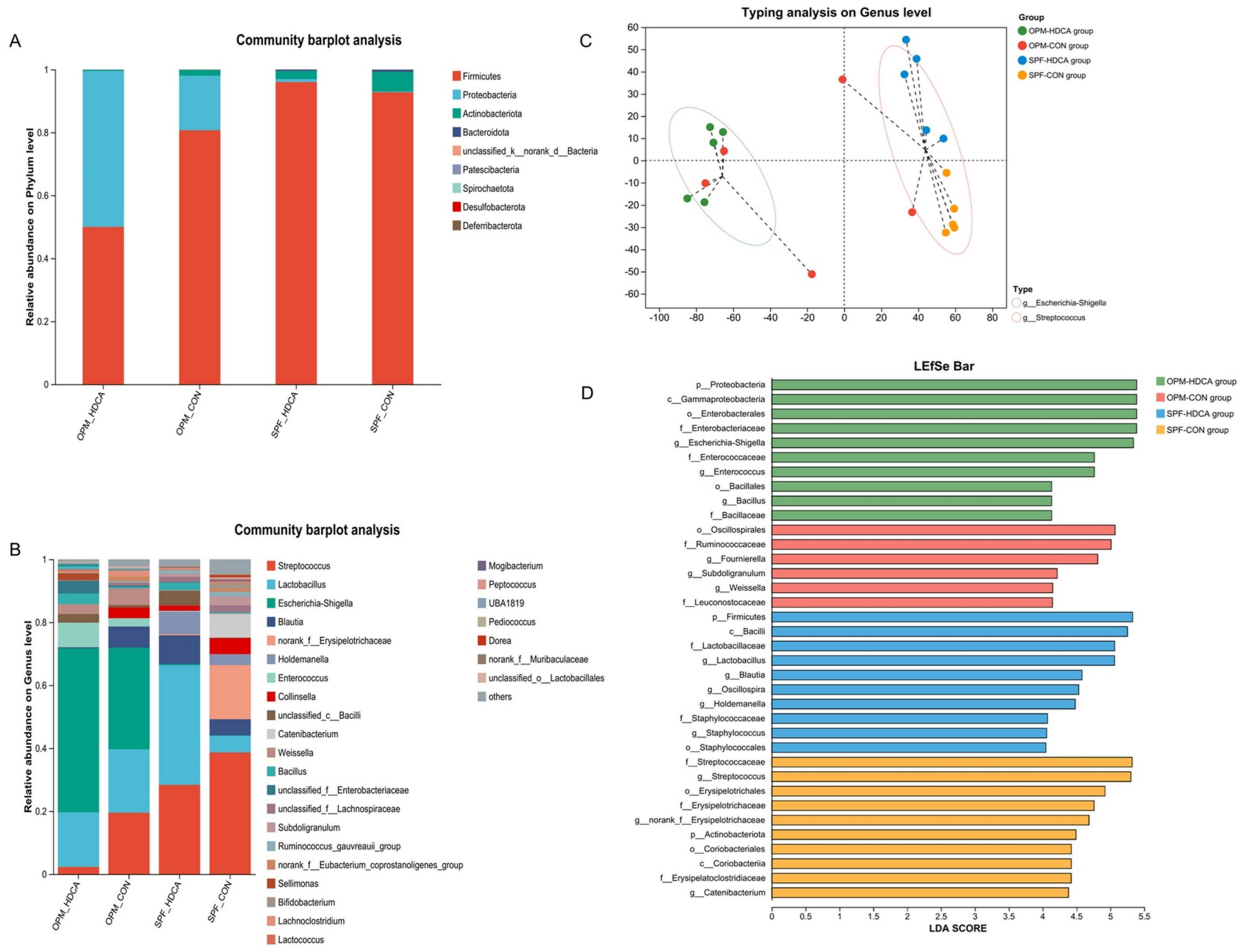
The impact of oral HDCA administration on intestinal microbiota remodeling in piglets. **(A)** The relative abundance of gut microbiota at the phylum level; **(B)** The relative of gut microbiota at the genus level; **(C)** Genus-level enterotype analysis; **(D)** LEfSe analysis bar chart.

Multiplexed single-end sequencing reads (1,648,383 in total) were imported into QIIME2, and finally resulted in 963,606 sequences ranging from 41,737 to 57,699 per sample (*n* = 20) representing 6,461 ASVs. Microbial community richness, as quantified by the Ace index at the ASV level, exhibited significant heterogeneity across the four experimental groups (Kruskal-Wallis H test, *p* = 0.022, shown in Supplementary Figure S1). Post-hoc analysis with Games-Howell multiple comparisons correction (*α* = 0.05) revealed two distinct differential patterns: the OPM-HDCA group showed significantly reduced species richness compared to both the SPF-HDCA group and SPF-CON group (*p* < 0.05), while no significant difference was detected compared to the OPM-CON group (*p* > 0.05) (shown in Supplementary Figure S1). In SPF-HDCA piglets, *Lactobacillus* abundance increased 7-fold (5.28% in SPF-CON group to 37.97%; *p* < 0.01), while *Streptococcus* decreased (28.34% vs. 38.65% in SPF-CON group; *p* < 0.05). OPM-HDCA piglets exhibited dominance of *Escherichia-Shigella* (52.13% vs. 32.23% in OPM-CON group; *p* < 0.01) and reduced *α*-diversity (Shannon index: 2.1 ± 0.3 vs. 3.8 ± 0.4 in OPM-CON group; *p* < 0.01) (shown in Figure 3).

Furthermore, as illustrated in Supplementary Figure S2, a statistically significant positive correlation was observed between the Gut Microbiota Health Index (GMHI) and the α-diversity index in the OPM piglets (Spearman correlation coefficient = 0.82, *p* < 0.05). Conversely, in the SPF piglets, the correlation between GMHI and the α-diversity index was notably weaker (Spearman correlation coefficient = 0.12, *p* > 0.05).

### 3.5 Enterotype-specific biomarkers

Results showed that all individuals in the OPM-HDCA group were classified as the *Escherichia-Shigella* enterotype, while all samples in the SPF-HDCA and SPF-CON groups were categorized as the *Streptococcus* enterotype (Figure 3C). LEfSe analysis identified significant biomarker microbial groups for each experimental group (shown in Figure 3B). In the OPM-CON group, key biomarkers included *Fournierella*, *Subdoligranulum*, and *Weissella*. For the OPM-HDCA group, the biomarkers shifted to *Escherichia-Shigella*, *Enterococcus*, and *Bacillus*. In the SPF-CON group, *Streptococcus* and *Catenibacterium* were notably prominent, whereas in the SPF-HDCA group, *Lactobacillus*, *Blautia*, *Oscillopira*, *Holdemanella*, and *Staphylococcus* (Figure 3D).

Additionally, bacterial genera with significant differences (LDA > 4, *p* < 0.01) were identified and used to construct an ASV set. A combined Receiver Operating Characteristic (ROC) curve analysis confirmed high diagnostic accuracy for distinguishing OPM-HDCA biomarkers from OPM-CON (AU value = 0.92), but poor discrimination between SPF-HDCA and SPF-CON (AU value = 0.60, Supplementary Figures S2C,D).

### 3.6 Intestinal barrier enhancement

**Figure 4:**
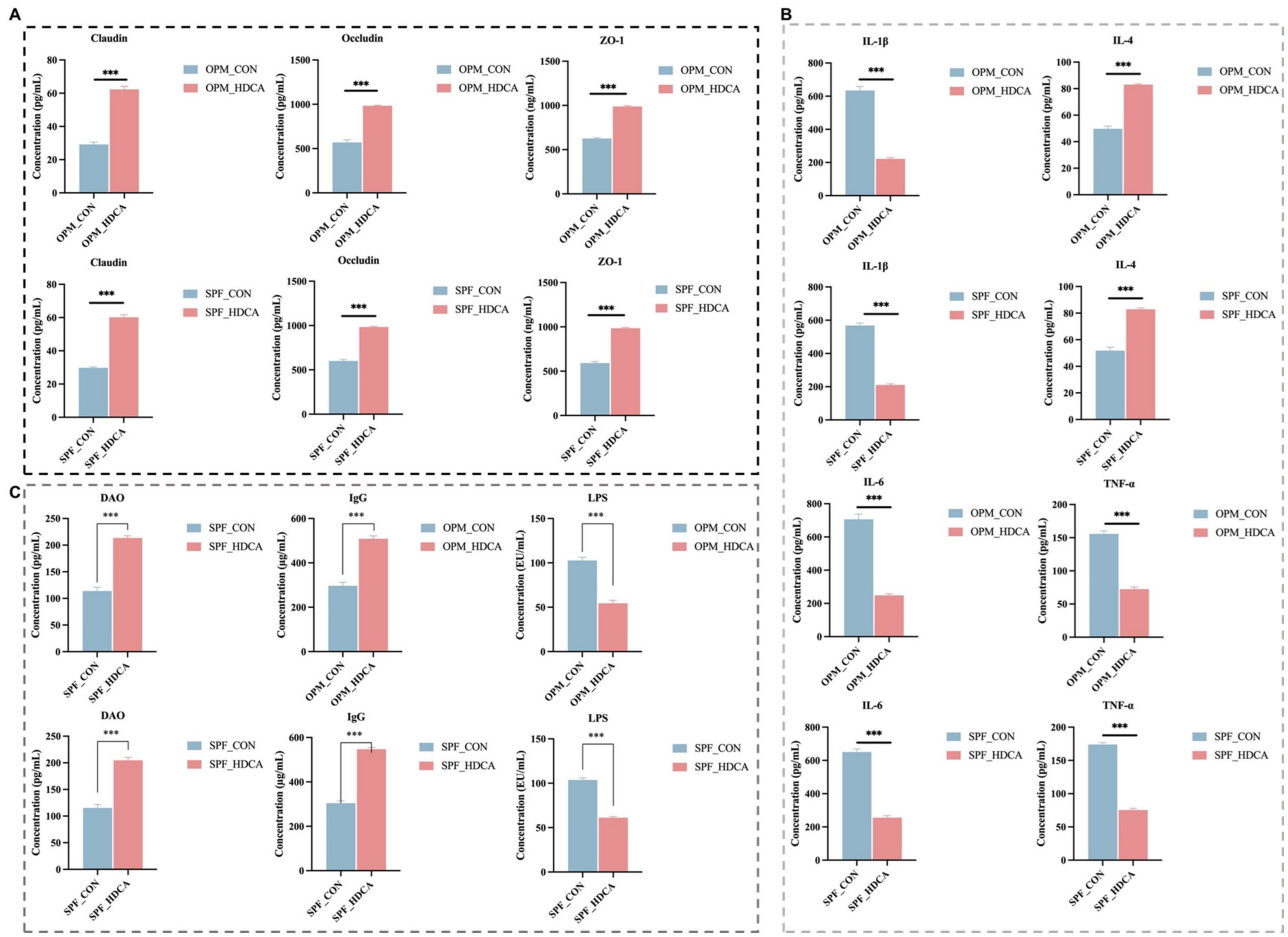
The impact of oral HDCA administration on intestinal barrier proteins and inflammatory markers in piglets. **(A)** Levels of tight junction proteins (ZO-1, Claudin, and Occludin); **(B)** Level of pro-inflammatory cytokines (TNF-α, IL-1β, and IL-6); **(C)** Levels of LPS, DAO and IgG. Significance: *p* < 0.05, *p < 0.01, **p < 0.001.

Oral HDCA administration significantly increased the levels of tight junction proteins (ZO-1, Claudin, and Occludin) in both OPM-HDCA and SPF-HDCA groups (*p* < 0.05, Figure 4A). Additionally, HDCA treatment significantly reduced pro-inflammatory cytokines (TNF-*α*, IL-1β, and IL-6) in ileal tissues (*p* < 0.05, Figure 4B). Serum analysis revealed that HDCA administration decreased LPS levels while increasing DAO and IgG levels compared to control groups (OPM-CON and SPF-CON, Figure 4C).

### 3.7 Bile acid metabolic reprogramming

HDCA elevated total serum bile acids (TBA) in OPM piglets (111.52 ± 14.00 μmol/L in OPM-HDCA group vs. 1.89 ± 1.19 μmol/L in OPM-CON group, *p* < 0.001). The primary conjugated bile acid glycocholic acid (GCA) and the secondary bile acid dehydrocholic acid (DHA) were undetectable in the colonic content samples of all SPF piglets (including both the SPF-CON and SPF-HDCA groups), but were present in all OPM piglets (including both the OPM-CON and OPM-HDCA groups). Additionally, the secondary conjugated bile acid glycoursodeoxycholic acid (GUDCA) was not detected in any OPM piglet samples, while the secondary conjugated bile acid glycochenodeoxycholic acid (GDCA) was only detected in the SPF-CON group samples. The secondary bile acid 12-ketolithocholic acid (12-KLCA) was detected in all groups except the SPF-HDCA group.

HDCA administration altered the conjugation patterns of bile acids (Supplementary Table S3). Specifically, the GCDCA/TCDCA ratio in the OPM-HDCA group was significantly lower than that in the OPM-CON group (*p* < 0.05). While the DCA/CA and UDCA/CA ratios also decreased in the OPM-HDCA group compared to the OPM-CON group following HDCA treatment, these changes did not achieve statistical significance due to high inter-individual variability within the groups (*p* > 0.05). In contrast, no GDCA was detected in the colonic contents of any individuals in the SPF-HDCA group after HDCA treatment, leading in GDCA/TDCA and GDCA/CA ratios of zero. Additionally, the GLCA/TLCA ratio in the SPF-HDCA group was significantly reduced (*p* < 0.05).

### 3.8 Gene expression modulation

**Figure 5:**
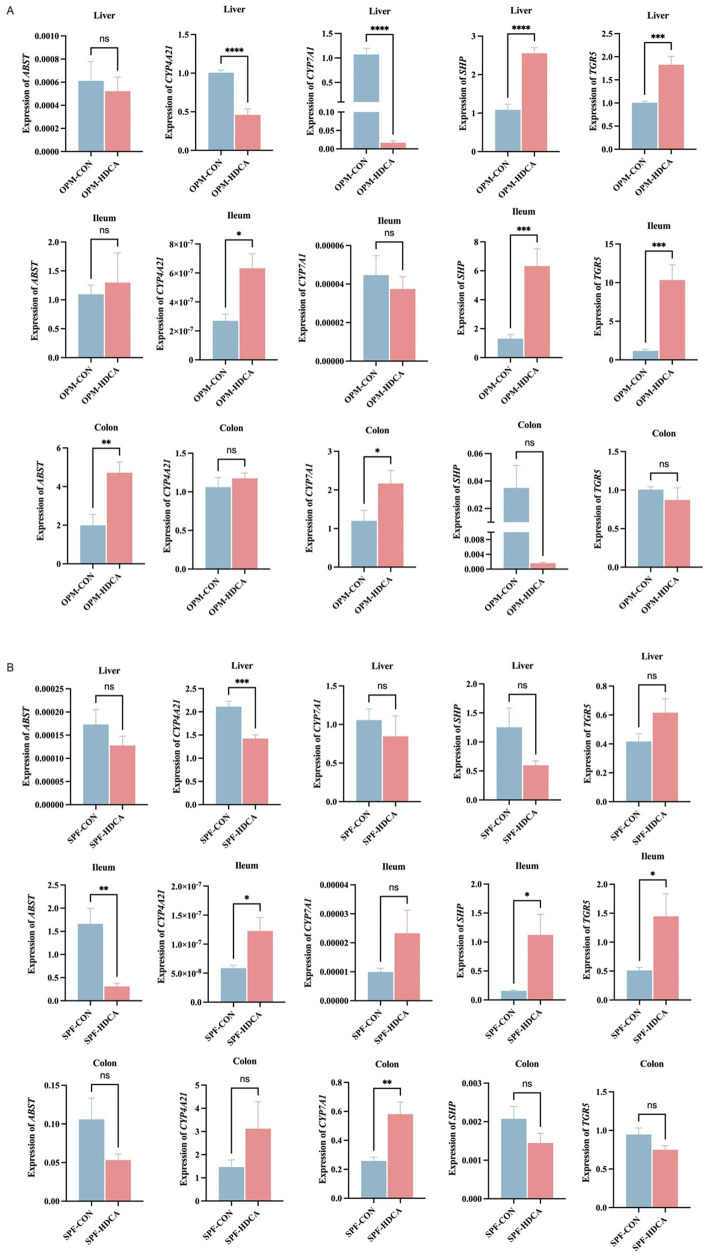
The impact of HDCA administration on the expressions of bile acid-related genes across gut microbiota environments. **(A)** OPM-CON group vs. OPM-HDCA group; **(B)** SPF-CON group vs. SPF-HDCA group.

HDCA differentially modulated the expressions of bile acid-related genes across gut microbiota environments (Figure 5). Compared to the OPM-CON group, the OPM-HDCA group demonstrated a significant reduction in the hepatic solute carrier family 10 Member 2 (*ASBT*) gene expression (*p* < 0.05) and a marked increase in hepatic *CYP4A27* gene expression (*p* < 0.001), while hepatic cytochrome P450 family 7 subfamily A Member 1 (*CYP7A1*) and small heterodimer partner (*SHP*) gene expressions were significantly downregulated (*p* < 0.001). In contrast, compared to the SPF-CON group, the SPF-HDCA group showed decreased expression of hepatic *ASBT*, *CYP7A1*, and *SHP* genes (*p* > 0.05), a notable reduction in hepatic cytochrome P450 family 4 subfamily A member 21 (*CYP4A21*) gene expression (*p* < 0.01), and an increase in Takeda G-coupled protein receptor (*TGR5*) gene expression (*p* > 0.05).

In the ileal and colonic tissues, HDCA treatment induced distinct patterns of bile acid-related gene expression. Compared to the OPM-CON group, *CYP4A27* expression was significantly upregulated in the ileum of the OPM-HDCA group (*p* < 0.05), but not in the colon (*p* > 0.05). This suggests that HDCA enhances *CYP4A27* expression specifically in the ileum within the OPM gut microbiota environment. Conversely, *CYP4A27* expression was significantly upregulated in both ileal and colonic tissues of the SPF-HDCA group compared to the SPF-CON group (Figure 5B). Similarly, *CYP7A1* expression was upregulated in both intestinal segments of the SPF-HDCA group relative to the SPF-CON group, suggesting a consistent effect of HDCA on *CYP7A1* expression. In the OPM-HDCA group, *SHP* and *TGR5* gene expression exhibited contrasting trends between ileal and colonic tissues. In the ileum, *SHP* and *TGR5* expression levels were significantly higher than those in the OPM-CON group (*p* < 0.001), whereas in the colon, expression was downregulated (*p* > 0.05). Under SPF conditions, the expression profiles of *SHP* and *TGR5* in ileal and colonic tissues paralleled those observed in the OPM groups.

## 4 Discussion

In the first study, the rapid gastrointestinal transit of HDCA in piglets (excretion within 5 h) contrasts sharply with its delayed absorption in GF mice (detected in the colon at 48 h). This disparity likely stems from differences in gastrointestinal physiology, such as gastric emptying rates and intestinal motility patterns between swine and rodents (20, 21). Notably, the absence of gut microbiota in GF mice may impair bile acid reabsorption via the enter-hepatic circulation, as microbial deconjugation of bile acids is essential for efficient *ASBT*-mediated uptake (12). In piglets, HDCA’s accelerated transit might reflect adaptive mechanisms in monogastric animals to optimize lipid digestion, consistent with the high basal bile acid concentrations observed in swine (6, 7). These findings underscore the necessity of species-specific models for evaluating bile acid kinetics, as rodent data may poorly extrapolate to swine—a critical consideration for translational nutrition research.

Specific bile acids, such as taurocholic acid (TCA) and *β*-taurine-conjugated muricholic acid (βTMCA), have been demonstrated to significantly promote the maturation of the intestinal microbiota in newborn mice, specifically by increasing the abundance of *Lactobacillus* and decreasing the abundance of *Escherichia* (22). The results of the second study suggest that the physiological effects of HDCA in piglet models are significantly dependent on the gut microbiota. In the OPM (naturally born and raised in a germ-free environment) piglets, HDCA at 0.2 mg/mL significantly suppressed growth (final body weight: 1.78 kg in OPM-HDCA group vs. 2.17 kg in OPM-CON group, *p* < 0.05). While in piglets that received FMT (naturally born and raised in a germ-free environment), this inhibitory effect was partially alleviated (2.82 kg in SPF-HDCA group vs. 3.13 kg in SPF-CON group, *p* > 0.05). This phenomenon is likely closely related to changes in the composition of the gut microbiota. For instance, in the SPF-HDCA group, the abundance of *Lactobacillus* significantly increased (37.97% vs. 5.28% in SPF-CON group), which may enhance mucus secretion and competitively inhibit pathogen colonization (23, 24). The bile salt hydrolases (BSHs) expressed by *Lactobacillus* can utilize bile acids as a nutrient source, thereby gaining a competitive advantage in the gut (25). In contrast, in OPM-HDCA group, *Escherichia-Shigella* genera were predominant (52.13% vs. 32.23% in OPM-CON group), and the proliferation of these pro-inflammatory bacteria may exacerbate intestinal barrier dysfunction and inflammation (15). Additionally, the study by Kuang et al. (2) revealed that HDCA significantly increased the abundance of the beneficial bacterium *Parabacteroides distasoni* by inhibiting intestinal farnesoid X receptor (FXR) activity in mice, while indirectly modulating the bile acid synthesis pathway, thereby effectively ameliorating NAFLD. In this study, HDCA treatment significantly activated the expression level of the TGR5 gene and also significantly upregulated the expression of tight junction proteins (ZO-1, Claudin, Occludin) in the ileum of piglets and inhibited pro-inflammatory cytokines (TNF-*α*, IL-1β), while increasing IgG levels and reducing LPS levels in piglet serum. These results indicate that HDCA exerts beneficial effects on the intestinal barrier of piglets, presumably by indirectly enhancing intestinal barrier function through activation of the *TGR5* signaling pathway, which is consistent with findings from mouse models (3, 8). This “host-microbiota” synergistic mechanism makes HDCA a potential intervention strategy for promoting early intestinal function maturation in piglets.

In terms of influencing the bile acid profile, HDCA administration drastically increased total serum bile acids in OPM piglets (111.52 μmol/L in OPM-HDCA group vs. 1.89 μmol/L in OPM-CON group), accompanied by downregulated *CYP7A1* and upregulated *TGR5* expression. This suggests HDCA suppresses *de novo* bile acid synthesis while enhancing receptor-mediated signaling—a feedback mechanism conserved across species (26). Intriguingly, the absence of GDCA in SPF-HDCA piglets implies microbial metabolism is essential for secondary bile acid formation, as GDCA typically arises from bacterial dehydroxylation of primary bile acids (12). The differential expression of *CYP4A27* (upregulated in OPM-HDCA ileum) and *CYP7A1* (downregulated in liver) highlights tissue-specific regulatory networks, possibly driven by microbiota-derived metabolites such as short-chain fatty acids (SCFAs), which modulate host epigenetic pathways (16).

### 4.1 Limitations and future directions

While this study provides critical insights, certain limitations warrant attention. First, HDCA at 0.2 mg/mL has a negative impact on the weight gain of piglets during lactation. Consequently, a dose–response study is necessary to identify optimal levels balancing growth and health outcomes. Second, the simplified microbiota in OPM/SPF models lacks the complexity of natural piglet microbiomes; future work should employ gnotobiotic models colonized with defined communities to dissect specific microbial taxa-HDCA interactions. Lastly, long-term trials are needed to assess HDCA’s effects on lipid homeostasis and immune tolerance in growing-finishing pigs.

## 5 Conclusion

This study demonstrates that HDCA exerts microbiota-dependent effects on growth performance, gut barrier function, and bile acid metabolism in neonatal piglets. Although 0.2 mg/mL HDCA treatment showed growth-suppressive effects in the suckling piglet model, it significantly improved intestinal health by increasing the abundance of beneficial gut microbiota (particularly *Lactobacillus* spp.), activating the TGR5 signaling pathway, and enhancing intestinal barrier function, demonstrating the importance of early-life gut microbiota environment in nutritional interventions. These findings advance our understanding of bile acid biology in livestock and provide a framework for developing precision nutrition protocols tailored to gut microbial ecosystems.

## Data Availability

The raw data supporting the conclusions of this article will be made available by the authors, without undue reservation.

## Footnotes

^1^ http://qiime2.org

## Supplementary material

SUPPLEMENTARY FIGURE S1 Microbial diversity and evenness (as measured by ASVs) in the ileum microbiota of newborn piglets. **(A)** Rarefaction curve (Observed Species, sobs); **(B)** Rarefaction curve (Shannon Index); **(C)** Ace index analysis. Data are presented as mean ± SD. Statistical significance: *0.01.

SUPPLEMENTARY FIGURE S2 The impact of oral HDCA administration on GMHI and its correlation with the Shannon index in the gut microbiota of piglets. **(A)** Comparison of GMHI between the OPM-HDCA and OPM-CON groups. **(B)** Comparison of GMHI between the SPF-HDCA and SPF-CON groups. **(C)** Spearman correlation between the Shannon index and GMHI in the OPM groups. **(D)** Spearman correlation between the Shannon index and GMHI in the SPF groups.

SUPPLEMENTARY FIGURE S3 Heatmap of correlation between the conjugated patterns of bile acids and blood biochemical indices across different groups.
