# Supplementary figures and images for: Hyodeoxycholic acid modulates gut microbiota and bile acid metabolism to enhance intestinal barrier function in piglets
Source: Front Vet Sci. 2025 Jun 20;12:1610956. doi: 10.3389/fvets.2025.1610956 (PMC12226288; doi:10.3389/fvets.2025.1610956)

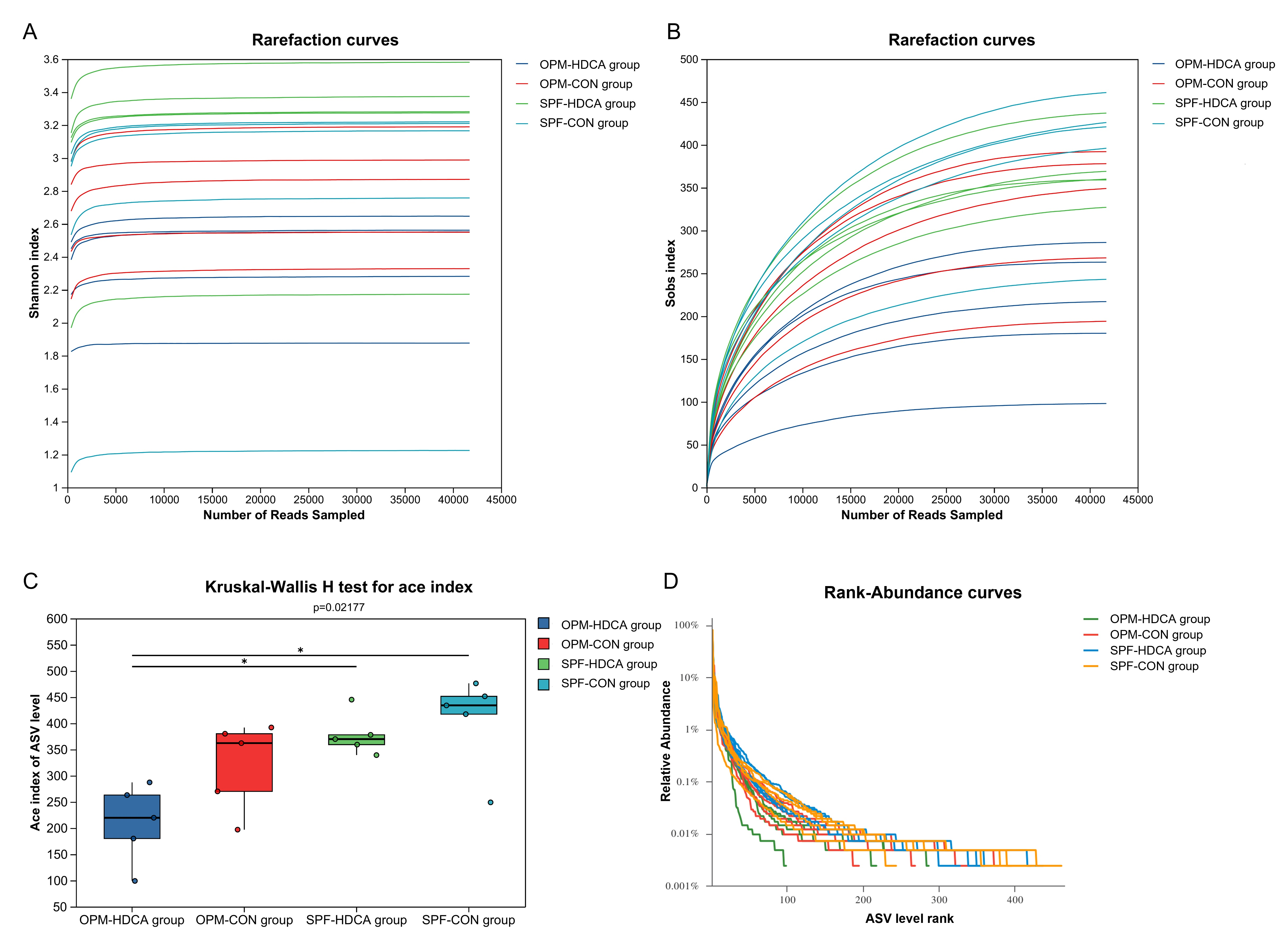

Supplement: SUPPLEMENTARY FIGURE S1 — Microbial diversity and evenness (as measured by ASVs) in the ileum microbiota of newborn piglets. (A) Rarefaction curve (Observed Species, sobs); (B) Rarefaction curve (Shannon Index); (C) Ace index analysis. Data are presented as mean ± SD. Statistical significance: *0.01. [file Image_1.tif]

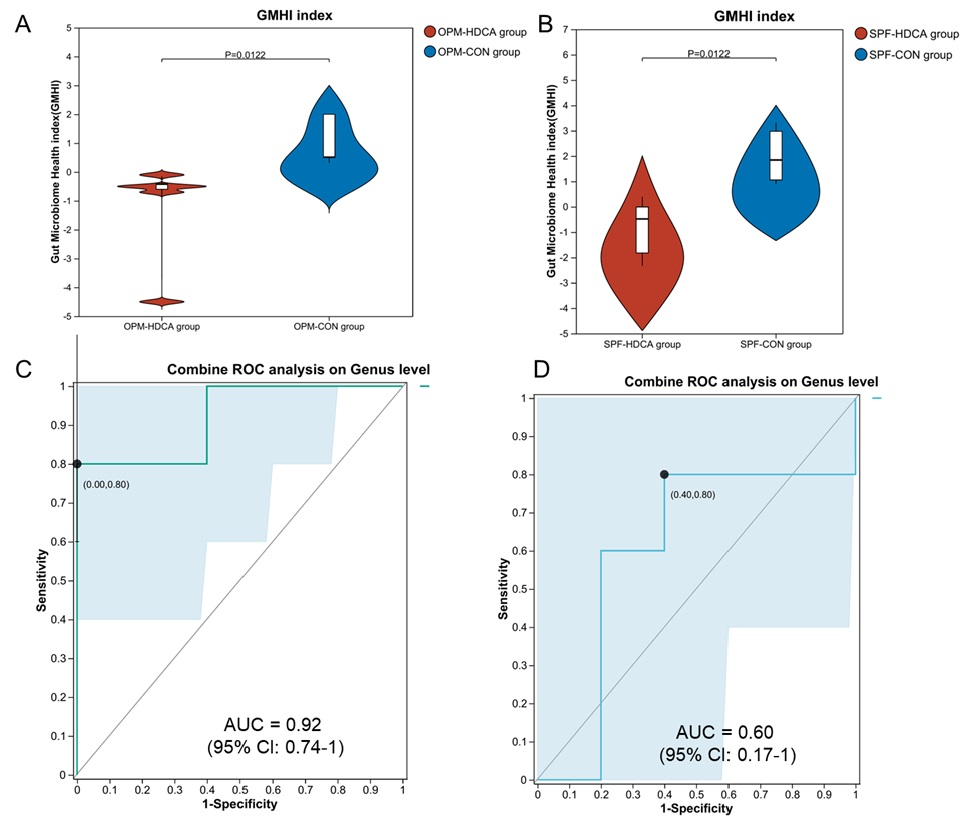

Supplement: SUPPLEMENTARY FIGURE S2 — The impact of oral HDCA administration on GMHI and its correlation with the Shannon index in the gut microbiota of piglets. (A) Comparison of GMHI between the OPM-HDCA and OPM-CON groups. (B) Comparison of GMHI between the SPF-HDCA and SPF-CON groups. (C) Spearman correlation between the Shannon index and GMHI in the OPM groups. (D) Spearman correlation between the Shannon index and GMHI in the SPF groups. [file Image_2.tif]

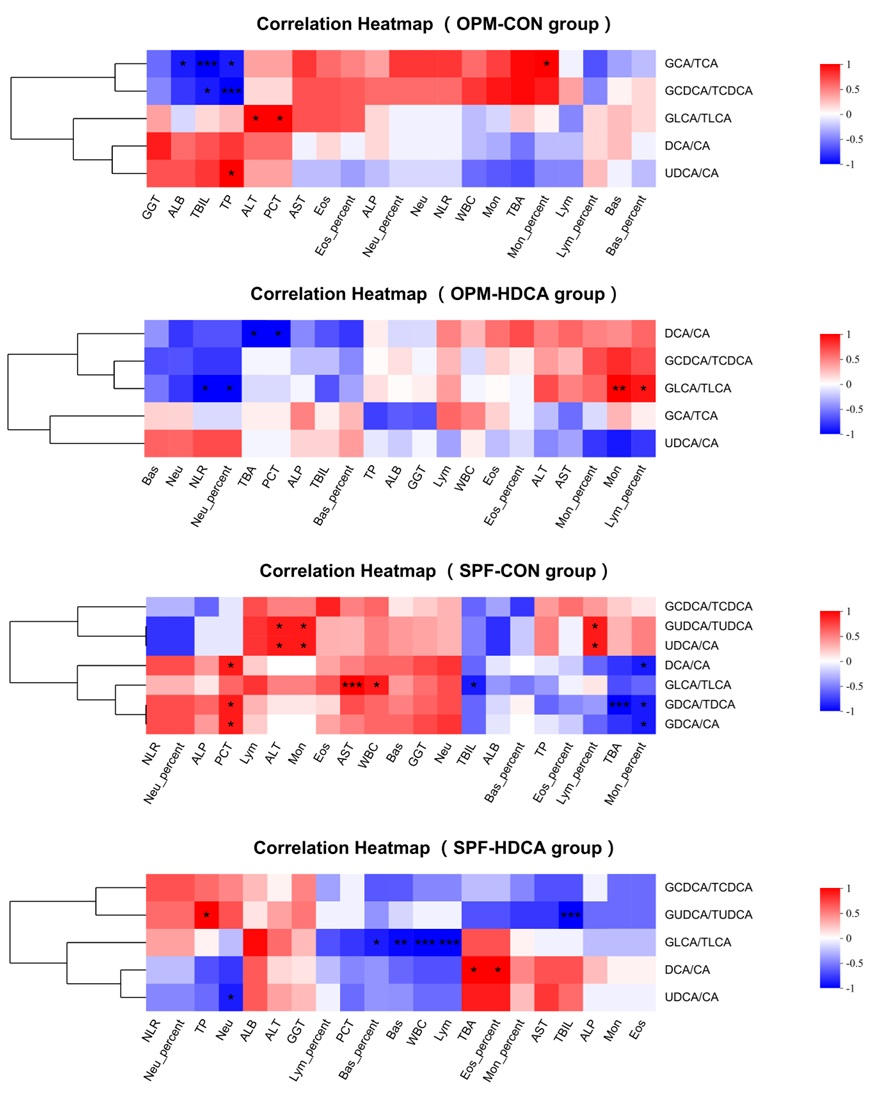

Supplement: SUPPLEMENTARY FIGURE S3 — Heatmap of correlation between the conjugated patterns of bile acids and blood biochemical indices across different groups. [file Image_3.tif]
