# Supplementary material for: Hyodeoxycholic acid modulates gut microbiota and bile acid metabolism to enhance intestinal barrier function in piglets
Source: Front Vet Sci. 2025 Jun 20;12:1610956. doi: 10.3389/fvets.2025.1610956 (PMC12226288; doi:10.3389/fvets.2025.1610956)
Supplement: Supplementary file 4 [file Table_1.docx]

Supplementary Table S1 Primer pairs involved in RT-qPCR quantitative analysis in this study.

| Gene | Gene description | Primer sequences (5’ to 3’) |
| --- | --- | --- |
| *TGR5* | G protein-coupled bile acid receptor 1 | Forward: TGCTGTCCCTCATCTCATTGG |
|  |  | Reverse: TGTGTAGCGATGATCACCCAG |
| *SHP* | Small Heterodimer Partner Nuclear Receptor | Forward: ACCATTCTCTTCAACCCTGATGT |
|  |  | Reverse: GCTGCGAGGAGAACACGAG |
| *ASBT* | Apical Sodium-Dependent Bile Acid Transporter | Forward: AAGTTCCTGGGGCACGTAAA |
|  |  | Reverse: CTCCTGGACAGCATCCCATT |
| *CYP7A1* | Cytochrome P450 Family 7 Subfamily A Member 1 | Forward: GAAAGAGAGACCACATCTCGG |
|  |  | Reverse: GAATGGTGTTGGCTTGCGAT |
| *CYP4A21* | Cytochrom P450 Family 4 Subfamily A Member 21 | Forward: GATCCAGGAACTGCATTGGGA |
|  |  | Reverse: CACAATTCCTTGAATAGGAACGGG |
| *GAPDH* | Glyceraldehyde-3-Phosphate Dehydrogenase | Forward: CCAGGGCTGCTTTTAACTCTG |
|  |  | Reverse: GTGGGTGGAATCATACTGGAACAT |

Supplementary Table S2 Comparison of bile acid composition and abundance (nmoL/g)^1^.

| BA type | OPM-HDCA group | OPM-CON group | *P*- value (OPM-HDCA vs OPM-CON) | SPF-CON | SPF-HDCA | *P*- value (SPF-CON vs SPF-HDCA) |
| --- | --- | --- | --- | --- | --- | --- |
| Glycocholic acid (GCA) | 0.0588±0.0730 | 0.0105±0.0145 | 0.215 | 0 | 0 | / |
| Glyco-chenodeoxycholic acid (GCDCA) | 0.4694±0.9343 | 0.1261±0.1154 | 0.438 | 0.0462±0.0200 | 0.0532±0.0382 | 0.726 |
| Tauro-cholic acid (TCA) | 0.2084±0.1804 | 0.0128±0.0039 | 0.072 | 0.0080±0.0125 | 0.0099±0.0041 | 0.756 |
| Tauro-chenodeoxycholic acid (TCDCA) | 2.6040±2.0425 | 0.0436±0.0330 | 0.049 | 0.0234±0.0137 | 0.0138±0.0053 | 0.185 |
| α-muricholic acid (α-MCA) | 1.7848±0.13957 | 0.5237±0.6638 | 0.106 | 2.9059±2.0870 | 1.1248±0.5182 | 0.101 |
| β-muricholic acid (β-MCA) | 11.0100±5.1879 | 2.6474±3.2719 | 0.016 | 2.8659±1.5123 | 3.0512±3.8747 | 0.924 |
| Tauro-α-muricholic acid (T-α-MCA) | 0.0341±0.0463 | 0 | 0.205 | 0.0127±0.0201 | 0 | 0.230 |
| Tauro-β-muricholic acid (T-β-MCA) | 0.1269±0.1206 | 0.0033±0.0024 | 0.084 | 0.0055±0.0043 | 0.0037±0.0030 | 0.468 |
| Cholic Acid (CA) | 8.6077±6.5137 | 2.1823±4.2581 | 0.102 | 0.8199±0.9170 | 1.5742±2.1531 | 0.501 |
| Chenodeoxycholic acid (CDCA) | 168.4211±118.8207 | 2.6018±5.1214 | 0.035 | 6.5997±9.3828 | 34.3068±47.6385 | 0.266 |
| CDCA-3-β-GA | 2.8129±1.5701 | 5.8071±3.6136 | 0.128 | 15.6858±11.7743 | 18.9612±5.8702 | 0.593 |
| Dehydrocholic acid (DHA) | 0.0045±0.0005 | 0.0041±0.0007 | 0.360 | 0 | 0 | / |
| Ursodeoxycholic acid (UDCA) | 9.8622±18.5777 | 0.8128±0.9875 | 0.338 | 12.4451±15.5632 | 22.0079±39.8024 | 0.630 |
| Glyco-ursodeoxycholic acid (GUDCA) | 0 | 0 | / | 0.0198±0.0159 | 0.0135±0.0259 | 0.655 |
| Allocholic acid (ACA) | 0.1553±0.1279 | 0.2070±0.2485 | 0.694 | 0.7290±1.0798 | 0.1406±0.1728 | 0.263 |
| Deoxycholic acid (DCA) | 0.5978±0.4300 | 0.8601±1.6183 | 0.735 | 11.6041±11.3801 | 0.8927±0.6307 | 0.103 |
| Tauro-ursodeoxycholic acid (TUDCA) | 0.0166±0.0041 | 0.0066±0.0078 | 0.035 | 0.0080±0.0029 | 0.0031±0.0032 | 0.033 |
| Hyodeoxycholic acid (HDCA) | 1527.4554±959.1371 | 10.0771±21.2919 | 0.024 | 262.8775±237.1344 | 913.4111±774.2234 | 0.136 |
| Apocholic acid (ApoCA) | 0.0210±0.0202 | 0.0015±0.0010 | 0.109 | 0.0648±0.0451 | 0.0938±0.1877 | 0.745 |
| Glyco-deoxycholic acid (GDCA) | 0 | 0 | / | 0.0121±0.0153 | 0 | 0.152 |
| Glyco-lithocholic acid (GLCA) | 0.0017±0.0017 | 0.0033±0.0050 | 0.535 | 0.0564±0.0625 | 0.0080±0.0110 | 0.127 |
| 7-Ketolithocholic acid (7-KLCA) | 18.7802±18.4221 | 1.0264±2.0642 | 0.065 | 6.9401±8.2342 | 7.0072±8.8752 | 0.99 |
| ω-muricholic acid (ω-MCA) | 3.5565±5.4958 | 18.7629±31.4758 | 0.318 | 89.3685±47.5933 | 111.6464±136.4590 | 0.744 |
| Murideoxycholic acid (мDCA) | 59.3049±48.0159 | 0.13900±0.1829 | 0.051 | 6.5151±5.6852 | 42.1896±27.4798 | 0.022 |
| Taurohyodeoxycholic acid (THDCA) | 16.7543±11.7447 | 0.0067±0.0072 | 0.033 | 0.0413±0.0419 | 0.0966±0.0455 | 0.008 |
| Taurohyocholic acid (THCA) | 2.6595±1.7863 | 0.0420±0.0319 | 0.031 | 0.0808±0.0642 | 0.0048±0.0063 | 0.03 |
| Taurolithocholic acid (TLCA) | 0.1978±0.1754 | 0.4475±0.2256 | 0.086 | 0.3223±0.3032 | 0.5202±0.2342 | 0.281 |
| Taurodeoxycholic acid (TDCA) | 0.0130±0.0108 | 0.0025±0.0011 | 0.097 | 0.0014±0.0005 | 0.0010±0.0004 | 0.166 |
| Lithocholic acid (LCA) | 1.5937±1.2064 | 4.2521±8.2854 | 0.498 | 77.6884±65.5748 | 13.6386±14.2786 | 0.094 |
| Hyocholic Acid (HCA) | 138.5066±82.1838 | 83.9005±106.1709 | 0.390 | 447.3059±234.4637 | 164.1995±223.3271 | 0.086 |
| Glycocholic acid (GHCA) | 1.4898±3.2310 | 0.3297±0.3156 | 0.468 | 0.7559±0.6004 | 0.2782±0.3212 | 0.155 |
| 23-Nor-Deoxycholic acid (23-Nor-DCA) | 0 | 0 |  | 0.0065±0.0042 | 0.0008±0.0016 | 0.021 |
| Isolithocholic acid (ILCA) | 0.0728±0.0692 | 0.7001±1.4068 | 0.375 | 28.9996±27.1628 | 4.4226±4.7955 | 0.081 |
| 12-Ketolithocholic acid (12-KLCA) | 0.0809±0.0629 | 0.0438±0.0763 | 0.426 | 2.1400±1.7190 | 0 | 0.05 |
| Dioxolithocholic acid (DLCA) | 0.0591±0.0587 | 0.5096±0.7546 | 0.253 | 11.0824±9.9487 | 2.0016±2.6365 | 0.111 |
| Lithocholic acid-3-sulfate (LCA-3-S) | 0.0771±0.1435 | 0.0063±0.0083 | 0.332 | 0.0309±0.0265 | 0.2808±0.1746 | 0.032 |
| 3β-Ursodeoxycholic acid (3β-UDCA) | 0.4386±0.6522 | 0.1883±0.2458 | 0.445 | 3.0568±3.2465 | 14.2501±23.7817 | 0.328 |
| 3-Dehydrocholic acid (3-DCA) | 0.1524±0.0839 | 0.4273±0.7973 | 0.488 | 0.0825±0.0704 | 0.0750±0.1004 | 0.896 |
| Chenodeoxycholic acid-24-Acyl Glycine (CDCA-24-AG) | 0.0072±0.0008 | 0.0055±0.0029 | 0.245 | 0.0064±0.0015 | 0.0079±0.0028 | 0.315 |
| 12-Ketochenodeoxycholic acid (12-KCDCA) | 3.5924±2.3291 | 2.4018±2.2280 | 0.433 | 0.3000±0.2361 | 3.8053±5.8049 | 0.248 |
| 7,12-Diethanolo lithocholic acid (7,12-DKLCA) | 0.0046±0.0021 | 0.0370±0.0650 | 0.328 | 0.0318±0.0205 | 0.0142±0.0078 | 0.131 |
| Ursodeoxycholic acid (UCA) | 0.0588±0.0664 | 0.7919±0.9514 | 0.160 | 2.1627±1.7178 | 2.4452±3.2380 | 0.869 |
| 7-Keto-Deoxycholic acid (7-KDCA) | 1.0241±0.6035 | 1.4072±2.6950 | 0.764 | 0.9019±0.8745 | 00.2210±0.0062 | 0.161 |
| Isodeoxycholic acid (IDCA) | 0.0080±0.0041 | 0.0068±0.0025 | 0.607 | 0.0124±0.0026 | 0.0121±0.0062 | 0.918 |
| 3β-Cholic acid (3β-CA) | 1.1189±0.6370 | 0.3481±0.4171 | 0.053 | 0.5624±0.4830 | 1.6147±0.6085 | 0.016 |
| Tauro-ω-muricholic acid (T-ω-MCA) | 0.4685±0.2044 | 0.0074±0.0052 | 0.007 | 0.0102±0.0123 | 0.0023±0.0025 | 0.195 |

^1^ SPF-HDCA group: piglets born naturally, raised in a germ-free environment, subsequently received fecal microbiota transplantation (FMT), and received 0.2 mg/mL HDCA orally; SPF-CON group: group: piglets born naturally, raised in a germ-free environment, subsequently received fecal microbiota transplantation (FMT), and received the equivalent volume of sterilized PBS solution orally as a control; OPM-HDCA group: piglets born naturally, raised in a germ-free environment without undergoing FMT, received 0.2 mg/mL HDCA orally; OPM-CON group: piglets born naturally, raised in a germ-free environment without undergoing FMT, received the equivalent volume of sterilized PBS solution orally as a control.

Supplementary Table S3 The impact of HDCA treatment on hematological parameters in piglets

| Hematological parameters | OPM-CON group | OPM-HDCA group | *P*-value (OPM-CON vs OPM-HDCA) | SPF-CON group | SPF-HDCA group | *P*-value (SPF-CON vs SPF-HDCA) |
| --- | --- | --- | --- | --- | --- | --- |
| white blood cell count (WBC), 10^9^/L | 8.40±1.52 | 6.61±0.62 | 0.51 | 12.00±2.43 | 7.20±1.08 | 0.08 |
| neutrophil percentage (NEU), % | 39.48±5.80 | 39.90±3.14 | 0.76 | 43.55±3.62 | 29.16±1.85 | *p*<0.01 |
| lymphocyte percentage (LYM), % | 53.03±6.24 | 54.67±3.20 | 0.97 | 45.79±3.57 | 59.46±2.15 | *p*<0.01 |
| monocyte count (MON), % | 5.93±1.04 | 4.18±0.97 | 0.30 | 7.91±0.64 | 8.68±0.60 | 0.39 |
| eosinophil count (EOS), % | 0.55±0.25 | 0.70±0.10 | 0.82 | 1.66±0.22 | 1.83±0.25 | 0.62 |
| basophil count (BAS), % | 1.03±0.43 | 0.55±0.12 | 0.16 | 1.09±0.07 | 0.88±0.11 | 0.15 |
| neutrophil count (NEU), 10^9^/L | 3.49±0.97 | 2.65±0.37 | 0.66 | 5.71±1.60 | 2.14±0.39 | 0.04 |
| lymphocyte count (LYM), 10^9^/L | 4.24±0.58 | 3.62±0.40 | 0.65 | 5.01±0.67 | 4.26±0.64 | 0.43 |
| monocyte count (MON), 10^9^/L | 0.55±0.18 | 0.27±0.06 | 0.24 | 0.97±0.23 | 0.62±0.10 | 0.16 |
| eosinophil count (EOS), 10^9^/L | 0.05±0.02 | 0.04±0.01 | 0.65 | 0.18±0.04 | 0.12±0.03 | 0.21 |
| basophil count (BAS), 10^9^/L | 0.08±0.03 | 0.03±0.01 | 0.10 | 0.13±0.03 | 0.06±0.01 | 0.05 |
| red blood cell count (RBC), 10^9^/L | 4.92±0.36 | 5.73±0.06 | 0.02 | 5.24±0.31 | 4.64±0.43 | 0.29 |
| hemoglobin concentration (HGB), 10^9^/L | 81.50±5.54 | 94.33±2.63 | 0.05 | 82.63±4.95 | 75.89±6.90 | 0.45 |
| hematocrit (HCT), % | 26.48±2.37 | 31.37±0.79 | 0.04 | 28.38±1.56 | 26.67±2.36 | 0.57 |
| mean corpuscular volume (MCV), fL | 53.50±1.29 | 54.82±1.48 | 0.43 | 54.36±1.08 | 57.79±0.97 | 0.03 |
| mean corpuscular hemoglobin (MCH), pg | 16.63±0.56 | 16.50±0.46 | 0.61 | 15.80±0.30 | 16.39±0.27 | 0.16 |
| mean corpuscular hemoglobin concentration (MCHC), g/L | 311.25±11.99 | 300.67±1.43 | 0.14 | 290.88±4.28 | 283.56±2.78 | 0.16 |
| red blood cell distribution width coefficient of variation (RDW-CV), % | 19.23±0.52 | 19.77±0.80 | 0.46 | 22.94±0.40 | 22.68±0.54 | 0.71 |
| red blood cell distribution width standard deviation (RDW-SD) | 42.00±1.01 | 44.27±1.20 | 0.09 | 51.71±1.43 | 54.38±1.58 | 0.24 |
| platelet count (PLT), 10^9^/L | 1146.50±598.14 | 779.67±36.83 | 0.32 | 753.13±146.33 | 599±144.48 | 0.47 |
| mean platelet volume (MPV), fL | 11.88±1.97 | 9.12±0.17 | 0.05 | 12.05±0.33 | 12.36±0.66 | 0.69 |
| platelet distribution width (PDW), % | 17.15±2.12 | 15.03±1.27 | 0.80 | 14.74±2.06 | 14.01±2.04 | 0.81 |
| plateletcrit (PCT), % | 1.73±1.12 | 0.71±0.04 | 0.18 | 0.91±0.19 | 0.75±0.18 | 0.56 |

Supplementary Table S4 The impact of HDCA treatment on biochemical parameters in piglets

| Biochemical parameters | OPM-CON group | OPM-HDCA group | *P*-value (OPM-CON vs OPM-HDCA) | SPF-CON group | SPF-HDCA group | *P*-value (SPF-CON vs SPF-HDCA) |
| --- | --- | --- | --- | --- | --- | --- |
| albumin (ALB), g/L | 3.14±0.30 | 3.23±0.07 | 0.75 | 2.83±0.05 | 3.04±0.06 | 0.01 |
| total protein (TP), g/L | 47.48±2.38 | 47.42±1.79 | 0.82 | 50.98±0.78 | 58.39±1.01 | *p*<0.01 |
| globulin (GLOB), g/L | 1.48±0.16 | 1.52±0.14 | 0.84 | 2.28±0.13 | 2.82±0.08 | *p*<0.01 |
| albumin/globulin ratio (ALB/GLB) | 2.33±0.38 | 2.22±0.16 | 0.81 | 1.12±0.06 | 1.10±0.04 | 0.68 |
| total bilirubin (TB), μmol/L | 7.33±2.04 | 9.05±1.61 | 0.25 | 1.73±0.15 | 9.91±2.16 | *p*<0.01 |
| gamma-glutamyl transferase (GGT), U/L | 72.25±5.04 | 81.83±15.29 | 0.50 | 53.88±4.46 | 147.78±32.32 | 0.02 |
| aspartate aminotransferase (AST), U/L | 50.75±15.31 | 59.67±5.46 | 0.69 | 58.25±3.00 | 151.33±20.38 | *p*<0.01 |
| alanine aminotransferase (ALT), U/L | 43.33±4.30 | 45.00±7.74 | ns | 37.75±2.79 | 74.56±10.12 | *p*<0.01 |
| alkaline phosphatase (ALP), U/L | 574.75±82.53 | 665.67±14.98 | 0.15 | 813.63±91.76 | 946.13±55.24 | 0.24 |
| TBIL, μmol/L | 2.19±0.63 | 99.62±6.60 | *p*<0.01 | 28.73±4.93 | 37.43±11.27 | 0.43 |
| amylase (AMY), U/L | 2343.75±251.66 | 2541.17±129.63 | 0.94 | 3096.00±114.45 | 2907.88±118.15 | 0.27 |
| lipase (LPS), U/L | 17.50±0.97 | 16.17±0.70 | 0.40 | 15.38±0.38 | 19.89±0.95 | *p*<0.01 |
| lactate dehydrogenase (LDH), U/L | 626.00±52.92 | 659±50.37 | 0.84 | 565.38±23.83 | 768.14±56.06 | *p*<0.01 |
| creatine kinase (CK), U/L | 255.75±624.12 | 286±22.61 | 0.32 | 170.75±10.97 | 664.56±204.70 | 0.04 |
| creatinine (CREA), μmol/L | 55.65±8.91 | 69.55±5.68 | 0.19 | 70.98±4.19 | 84.41±2.55 | 0.01 |
| blood urea nitrogen (BUN), mmol/L | 5.06±0.31 | 4.36±0.36 | 0.23 | 4.59±0.24 | 7.06±0.42 | *p*<0.01 |
| urine protein-to-creatinine ratio (uCRE) | 113.89±30.88 | 65.16±7.36 | 0.17 | 65.23±2.94 | 83.63±4.09 | *p*<0.01 |
| blood glucose (GLU), mmol/L | 5.64±0.61 | 4.94±0.37 | 0.42 | 5.32±0.85 | 5.27±0.44 | 0.96 |
| total cholesterol (TCH), mmol/L | 3.76±0.40 | 3.07±0.27 | 0.39 | 2.87±0.12 | 3.13±0.16 | 0.22 |
| triglycerides (TG), mmol/L | 1.37±0.48 | 1.24±0.15 | 0.94 | 1.04±0.17 | 0.76±0.11 | 0.24 |
| total carbon dioxide (TCO_2_), mmol/L | 20.38±1.55 | 16.40±1.45 | 0.17 | 20.63±0.54 | 19.59±0.63 | 0.24 |
| calcium (Ca), mmol/L | 2.68±0.10 | 2.75±0.04 | 0.48 | 2.64±0.05 | 2.81±0.05 | 0.02 |
| inorganic phosphorus (PHOS), mmol/L | 4.60±0.40 | 4.02±0.10 | 0.24 | 4.33±0.17 | 5.52±0.17 | *p*<0.01 |
